# Clinical characteristics and thromboembolic risk of atrial fibrillation patients with and without congestive heart failure. Results from the CRATF study: Erratum

**DOI:** 10.1097/MD.0000000000018071

**Published:** 2019-11-11

In the article, “Clinical characteristics and thromboembolic risk of atrial fibrillation patients with and without congestive heart failure. Results from the CRATF study”,^[1]^ which appears in Volume 97, Issue 45 of *Medicine*, the CRAFT was misspelled in the title as CRATF.
